# A Novel Setup and Protocol to Measure the Range of Motion of the Wrist and the Hand

**DOI:** 10.3390/s18103230

**Published:** 2018-09-25

**Authors:** Kostas Nizamis, Noortje H. M. Rijken, Ana Mendes, Mariska M. H. P. Janssen, Arjen Bergsma, Bart F. J. M. Koopman

**Affiliations:** 1 Department of Biomechanical Engineering, Technical Medical Centre, University of Twente, Drienerlolaan 5, 7522 NB Enschede, The Netherlands; amendes06@hotmail.com (A.M.); a.bergsma@utwente.nl (A.B.); h.f.j.m.koopman@utwente.nl (B.F.J.M.K.); 2 Department of Rehabilitation, Donders Institute for Brain, Cognition and Behaviour, Radboud University Medical Centre, Reinier Postlaan 4, 6525 GC Nijmegen, The Netherlands; n.h.rijken@utwente.nl (N.H.M.R.); Mariska.Janssen@radboudumc.nl (M.M.H.P.J.)

**Keywords:** distal upper extremities, goniometer, hand, leap motion sensor, motion capture, range-of-motion, rehabilitation, wrist

## Abstract

The human hand is important for the performance of activities of daily living which are directly related to quality of life. Various conditions, such as Duchenne muscular dystrophy (DMD) can affect the function of the human hand and wrist. The ability to assess the impairment in the hand and the wrist by measuring the range of motion (ROM), is essential for the development of effective rehabilitation protocols. Currently the clinical standard is the goniometer. In this study we explore the feasibility and reliability of an optical sensor (Leap motion sensor) in measuring active hand/wrist ROM. We measured the hand/wrist ROM of 20 healthy adults with the goniometer and the Leap motion sensor, in order to check the agreement between the two methods and additionally, we performed a test-retest of the Leap motion sensor with 12 of them, to assess its reliability. The results suggest low agreement between the goniometer and the leap motion sensor, yet showing a large decrease in measurement time and high reliability when using the later. Despite the low agreement between the two methods, we believe that the Leap motion sensor shows potential to contribute to the development of hand rehabilitation protocols and be used with patients in a clinical setting.

## 1. Introduction

The human hand is one of the most complex and versatile anatomical structures in the human body [1] and plays an important role in a person’s ability to interact with the environment. Reduced functioning of the hands may occur for example as a consequence of increasing age [2], traumatic injuries such as amputation of a finger or thumb [3,4], diseases on the nervous system like carpal tunnel syndrome, stroke and Parkinson’s disease [5], or diseases that affect the muscle, like in neuromuscular diseases [6]. Hand function impairments can restrict the independence of the affected individual and thus quality of life [4,7]. To evaluate the level of dysfunction and to guide adequate therapeutic strategies, reliable assessment of hand function and joint range of motion (ROM) is important [8]. 

We are specifically interested in disabilities of the upper extremity related to Duchenne Muscular Dystrophy (DMD). DMD is a neuromuscular disease affecting around 1:5000 male births worldwide [9]. Mutations in the dystrophin gene, lead to progressive muscular weakness and disability due to loss of skeletal muscle strength [10]. This includes the muscles in the forearm that control the movements of the hand and wrist and first signs of muscle loss are already visible in the late ambulatory stage [11]. Lately, interventions are proposed for assisting the hand function of people with DMD such as passive hand/wrist orthoses [12]. These are complimentary to physical therapy already aiming to preserve as much functionality as possible [13]. In the Flextension-Symbionics project we are currently developing an active hand exoskeleton in order to actively assist the hand function of people with DMD [14]. 

For the measurement of ROM in clinical practice, goniometry is widely used [8]. Different types of joints require different types of goniometers, in terms of size and shape. In general, goniometers are low-cost, lightweight and portable. However, its intra-rater reliability depends on the experience of the rater and interrater reliability is quite low [15] and not consistent over time [16]. With a goniometer, only one joint at a time can be measured, making the procedure for the whole hand time-consuming for both the rater and the participant [17].

Throughout the last years, new techniques have been developed that enable dynamic analysis of kinematics of the hands. Such techniques may have potential for objective clinical evaluation of the ROM of hand and fingers. Motion tracking devices can measure dynamic parameters through cameras [18], gloves [7,19,20,21] or by attaching sensors to the skin of the user [22,23]. However, most of these instruments are expensive, take a lot of time to setup or are difficult to don and doff by people with hand deformities or severe muscle weakness, like in DMD. Pham et al. developed a non-contact camera based system that showed very promising results for the tracking of the finger MCP, PIP and DIP joints [24]. However, the price of that camera is still relatively high and the proposed system is not currently commercially available. 

The Leap motion sensor (Leap Motion Inc., San Fransisco, CA, USA) is able to detect hand kinematics through the use of three infrared emitters and two small camera’s incorporated in one sensor. It already has a wide range of applications related to hand gesture recognition, such as manipulation of robots [18], human-computer interfaces [25] and gaming [26]. Because the Leap motion sensor does not require contact with the individuals’ hands or the use of markers, assessments can be performed fast. Furthermore, it is a low cost solution and it can minimize significantly measurement time due to the fact that more than one joint can be measured simultaneously. It has been indicated that due to internal constraints of hand angles estimation, the leap is not a promising sensory modality for clinical practice [24]. However, a recent study showed promising results for the finger MCP joints using the Leap motion sensor [27].

In this study, we propose a new clinical assessment protocol using off the shelf, low cost components, namely the Leap motion sensor and Brekel software (Pro Hands 1.27, Brekel, Amsterdam, Noord Holland, The Netherlands). We measured the maximal active voluntary angles of fingers, thumb and wrist in twenty healthy participants with no hand impairments. The goal of this study is to evaluate the accuracy and reliability of the Leap motion sensor for measuring hand and wrist ROM by (1) comparing the active ROM of the wrist, hand and fingers measured using the Leap motion sensor to goniometer measurements and (2) determine the test-retest reliability of the Leap motion ROM measurements.

## 2. Materials and Methods

### 2.1. Study Participants

Twenty healthy persons participated in the study (all right-handed, 20–26 years old, eight males and twelve females). None of the participants had previous traumas (e.g., bone fractures) of their hands or fingers. Twelve of the participants, were re-measured with the Leap motion sensor. The Medical Ethics Committee of Twente decided that this study does not require a medical ethical approval (K17-41). The study was conducted according to the ethical standards given in the Declaration of Helsinki of 1975, as revised in the year 2008. All participants were informed via a letter and signed a consent form prior to the experiment.

### 2.2. Materials and Data Acquisition

**Figure 1:**
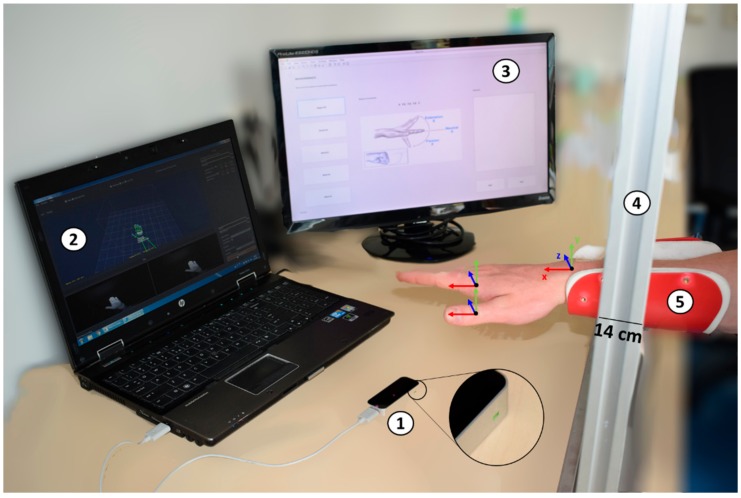
Experimental setup. (1) The controller of the Leap sensor. The green light indicates that the controller is on. (2) Brekel Pro Hands application in real-time, on the host computer. (3) The Guide User Interface shows which movement the participant should do. (4) The platform with (5) arm support at a distance of 14 cm from the table’s surface, where the participant should place his forearm.

The measurement setup consists of four components. The Leap motion sensor, a software package to obtain and record the hand and wrist ROM from the Leap motion sensor (Brekel Pro Hands 1.27), a Matlab-based graphical user interface to instruct the participants on how to perform the movements and analyze the data and a mechanical setup for positioning the arms of a person (Figure 1).

The Leap motion sensor is a low-cost consumer-grade camera system with three infrared emitters and two cameras. Data from the Leap are recorded with a rate of up to 300 frames per second (fps). The Leap sensor includes a controller and it has its own coordination system and skeletal model of the human hand. It has a field of view of about 150 degrees and approximately up to 600 mm above the device, which enables 3D tracking of the hands. 

Based on a pilot measurement, we determined the following conditions to be optimal for Leap motion use: The distance between the controller and the participant’s hands, and the orientation of the hand itself, are crucial to avoid occlusion and aliasing. The best performance was achieved when the distance above the sensor was kept between 14 and 24 cm, which is also in accordance with previous literature [28]. The suggested starting finger configuration is with spread fingers [29]. However, people with DMD may have difficulties spreading the fingers. We found no influence of the starting finger configuration on the quality of the recorded data. Regarding the light, optimal recordings were achieved when the artificial light in the room was switched off and the curtains were closed. Regarding jewelry, watches and clothing, the most optimal recording quality was achieved when the arms were uncovered. 

To record the data from the leap sensor, the software application Brekel Pro Hands v1.27’ [29] was used. This application enables recording of motion of up to two hands and forearms using a Leap controller. The displayed data distinguish the left and right side and the position and orientation values for each joint and fingertips of the digits, elbow, wrist and palm are recorded in 3D. 

To check and save the data provided by the Brekel application of each participant and also to serve as a visual cue for the participant with respect to the movement they had to perform, we created a Graphical User Interface (GUI) in MATLAB (R2016b, The MathWorks, Inc., Natick, MA, USA), presented in Figure 1. The raw data of the orientation of the joints were stored in separate *.csv files which were stored in Matlab by the GUI as *.mat files. Data were captured with a rate of 115 fps. A low-pass 2nd order Butterworth filter with a cutoff frequency of 2 Hz, similar to the one used by Lanari Bó et al. [30] was used to smooth the raw joint angle data. 

A setup with arm supports was constructed to allow participants to rest their arms above the Leap sensor (Figure 1). The positioning of the arm support is adjustable vertically and horizontally. The preferred forearm of the participant was placed on the arm support. The arm support was set such that the lower arm of the participant was resting in a comfortable position, and the hand was placed above the Leap motion sensor in the center of the body. The setup allowed for the unrestricted completion of the wrist, fingers and thumb. Since the movements of the joints are with respect to the local coordinate frame (Figure 1) performed in 2D, only rotation values of one axis were considered for each joint. The angles of the joints were measured by the Brekel software around the local *x*,*y*,*z* axes of every joint (Figure 1). 

### 2.3. Experimental Protocol

**Figure 2:**
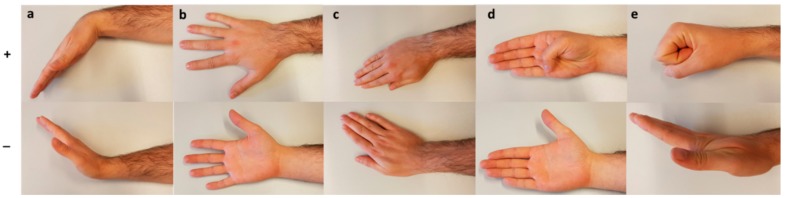
Representation of one trial of each movement the participants need to perform. All movements start at the neutral position. The movements are: (**a**) flexion and extension of the wrist; (**b**) radial and ulnar deviation of the wrist; (**c**) pronation and supination of the wrist; (**d**) flexion and extension of the MCP and IP joints of the thumb; and (**e**) flexion and extension of the MCP, PIP and DIP joints of the four fingers.

For assessments with the Leap, from the initial resting position, participants were asked to actively move their fingers and maximally perform one by one the following five movements with their hand and wrist: flexion/extension of the fingers (MCP, PIP and DIP) by making a fist and then extending, flexion/extension of the thumb (MCP and IP), by flexing the thumb maximally in a plane parallel to the palm and subsequently try to touch the palmar side of the little finger’s MCP, radial/ulnar deviation of the wrist, pronation/supination of the forearm and flexion/extension of the wrist (Figure 2). The participants had to repeat each movement three times, while resting their arm on the arm support (Figure 1). The mean of the three repetitions was used for the data analysis. 

**Figure 3:**
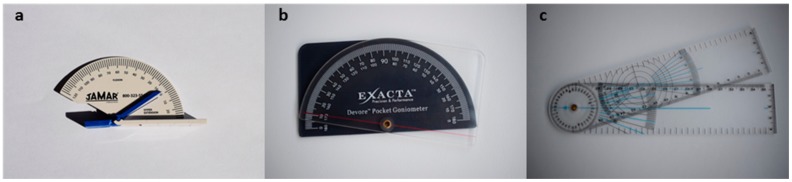
Goniometers used in this study: (**a**) Rolyan finger goniometer with loose-fitting hinge; (**b**) Devore pocket goniometer; and (**c**) universal goniometer with full-circle body.

To obtain the angles manually with the goniometer, the raters of this study were trained by an experienced clinical evaluator to measure the angles for the different joints. Three different goniometers, which varied in size, were used to measure the joint angles (measured in degrees). To measure the angles of the DIP joint of the fingers and IP joint of the thumb, a plastic Rolyan finger goniometer with loose-fitting hinge (Figure 3a) was used. It has a resolution of 2° increments, and ranges from 30° of hyperextension to 120° of flexion. To measure the angles of the MCP and PIP joints of the fingers and MCP joint of the thumb, a plastic Devore pocket goniometer (Figure 3b) with a resolution of 1° increments, and a reading range of 180° was used. To assess the wrist joint, a plastic universal goniometer with full-circle body (Figure 3c) with a resolution of 2° increments was used.

All 20 participants performed one measurement with the Leap and one with the goniometer, consisting of three repetitions per measuring methods. Additionally, 12 of them performed an additional measurement (also with three repetitions) with the Leap sensor two weeks after the first one. This was done to assess the test-retest reliability of the Leap measurements. Flexion/extension of a total of 14 finger joints (MCP, PIP and DIP for the four digits and MCP and IP of the thumb) and three degrees of freedom of the wrist (pronation/supination, radial/ulnar deviation and flexion/extension) were measured with each measurement methods (Figure 3). All flexion angles, together with pronation and radial deviation were taken as positive, while extension angles, ulnar deviation and supination were taken as negative. The accuracy of the evaluation protocol was defined as the agreement between the Leap motion sensor and the goniometer. The reliability of the assessment was defined as the consistency of the measure with repeated observations by the Leap motion sensor.

The time-consumption (in minutes) is recorded for both techniques as the amount of time that was needed to perform the measurement (including the three repetitions) for each method. The time that was needed for preparation of the participant and the setup was not considered in this. was not considered in this. The time was measured using a stopwatch. 

### 2.4. Statistical Analyses

Minimum and maximum active joint angles measured with Leap motion sensor and goniometer are compared using Bland-Altman plots and quantified by the mean difference and limits of agreement. Compared to previous similar studies [24], we avoided the use of Intraclass Correlation Coefficient (ICC) analysis. Our choice was motivated by the fact that there are no standard values for acceptable reliability using ICC [31] and that we had low variability in maximum flexion and extension angles between our participants, which disables the use of correlational analysis. It has been suggested that correct use of ICC as a rule of thumb includes the acquisition of at least 30 heterogeneous samples when conducting a reliability study [31]. We first calculated the difference of the mean between the Leap motion sensor and the goniometer and, between test and retest for every individual joint. One sample *t*-tests were performed to check if these differences of means differ significantly from zero. Differences of means were normally distributed. Only 8 out of the 68 differences, moderately violated the assumption of normality, as assessed by Shapiro-Wilk’s test (*p* < 0.05. However, the one sample *t*-test is quite robust to moderate violations of normality [32]. The statistical analysis was performed with IBM SPSS v24 (IBM, Armonk, NY, USA).

## 3. Results

### 3.1. Evaluation of the Leap Motion Sensor

**Figure 4:**
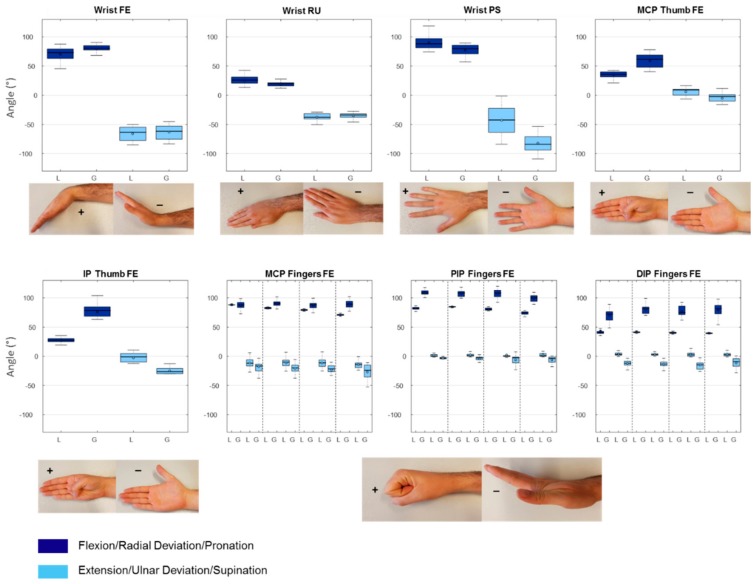
Boxplots of the maximal and minimal values of all the joints measured with the Leap motion sensor and the goniometer. L: Data from the Leap motion sensor; G: Data from the goniometer; FE: Flexion/Extension; RU: Radial/Ulnar deviation; PS: Pronation/Supination; For the fingers, the order is index, middle, ring, little, from left to right. For every pair of boxplots, flexion/radial deviation/pronation is left and extension/ulnar deviation and supination right.

All participants performed all the movements and all data were collected successfully with both the Leap sensor and the goniometer. Mean values and standard deviations of both the goniometer and Leap measurements can be found in Table 1, together with the mean difference between measurements. One sample *t*-test *p*-values for comparing the mean differences to zero for every joint measured are reported in Table 1. Results are also displayed graphically with boxplots (Figure 4). From this figure it becomes clear that for the wrist, the leap and goniometry results are quite comparable. MCP angles are also comparable but differences seem to increase when moving from index to little finger and from proximal to distal joints. Overall, the mean flexion angles of most joints are underestimated when measured with the Leap motion sensor. Furthermore, all finger joints showed smaller standard deviations for the Leap motion sensor results compared to the goniometer results. Maximum extension measured with the goniometer reveals negative values for all joints, which indicate hyperextension. Leap results also show hyperextension in some of the joints, but most extension angles are less extreme compared to the goniometer results. When measured with the leap, DIP and PIP joints never go below zero.

**Figure 5:**
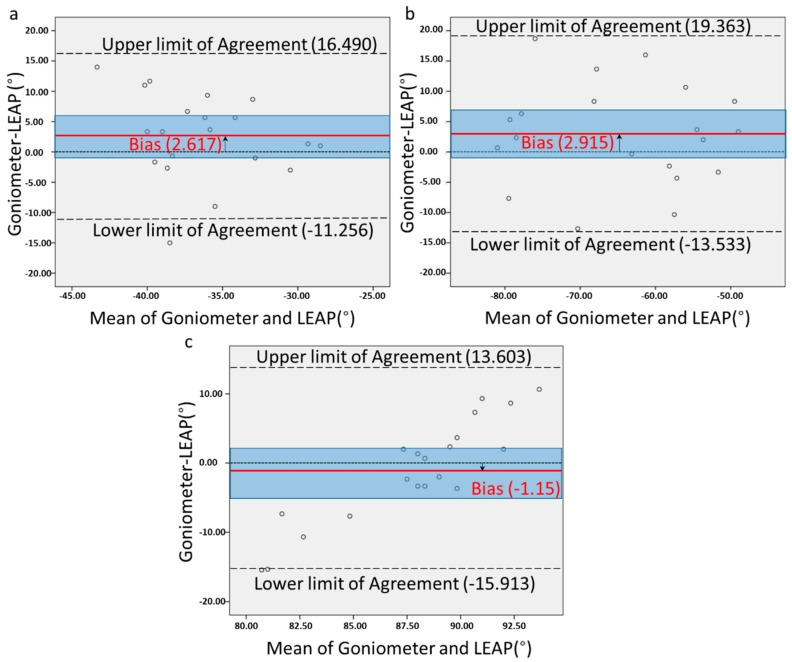
Bland-Altman plots of (**a**) wrist ulnar deviation, (**b**) wrist extension and (**c**) Index finger MCP flexion. The y-axis shows the difference, while the x-axis the mean between goniometer and Leap motion sensor for every participant (20 participants/dots). The red line shows the mean difference (bias) for the two measurement techniques. The blue shaded area is the 95% CI for the mean difference. For these three movements the line of equality (line crossing zero) is included inside the shaded area. The two dashed lines show the limits of agreement (±1.96*SD), between the two measurement techniques.

Based on the statistical analysis, we found satisfactory agreement between the goniometer and the Leap motion sensor only for three movements. These are wrist extension, index MCP flexion and ulnar deviation. In Table 1, it is shown that for these movements the 95% CI for the mean difference is small and it includes zero (Figure 5). The one sample *t*-test *p*-values, for these three movements do not show a statistically significant difference for the mean difference between the goniometer and the Leap motion sensor from zero.

### 3.2. Test-Retest

**Figure 6:**
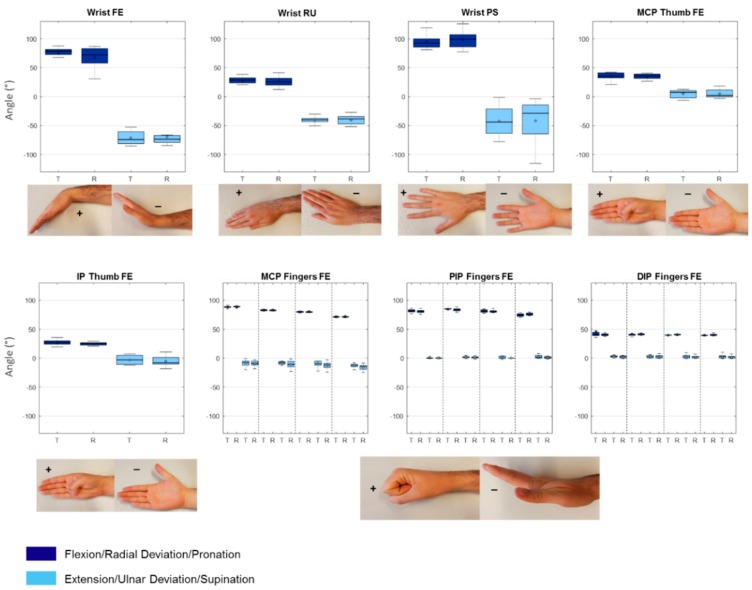
Boxplots of the maximal and minimal values of all the joints measured with the Leap motion sensor test and retest. T: Test data from the Leap motion sensor; R: Retest data from the Leap motion sensor; FE: Flexion/Extension; RU: Radial/Ulnar deviation; PS: Pronation/Supination; for the fingers, the order is index, middle, ring, little, from left to right. For every pair of boxplots, flexion/radial deviation/pronation is left and extension/ulnar deviation and supination right.

Test-retest assessment of Leap measurements was done with 12 of the 20 participants. The results are reported in Table 2 and visualized with boxplots in Figure 6. During the retest assessments, again small standard deviations were found, especially for finger joint flexion results. The statistical analysis revealed that for all movements assessed, small differences were present between test and retest values. Furthermore, for all movements except little finger DIP flexion, a difference of zero was within narrow 95% CI of the mean difference. Broader 95% CI were found for pronation/supination and wrist flexion/extension, however still including zero within them.

### 3.3. Measurement Time

Using the goniometer took on average 32:65 min per participant, with a standard deviation of 10:86 min. With the use of the Leap motion sensor, the rater was able to measure on average every participant within 7:22 min, with a standard deviation of 2:47 min.

## 4. Discussion

### 4.1. Rationale

In this study we aimed to evaluate the accuracy and reliability of the Leap motion sensor together with a novel protocol, to measure the ROM of the wrist and finger joints. To the authors’ best knowledge, this is the first study to assess accuracy and test-retest reliability of this system for hand and wrist joint angles measurement. This was done by comparison of the Leap motion sensor to the current clinical standard for hand and wrist angle measurements; the goniometer. Additionally, test retest reliability of the Leap motion sensor was examined. Using goniometry as the golden standard for evaluating the accuracy of the Leap motion sensors is questionable. Other high precision optical techniques, such as cameras with reflective markers could be used for a more meaningful comparison. However, goniometry is the current clinical standard, and there is a plethora of studies related to measurements, protocols and different goniometers [15]. Moreover, comparable studies evaluated new and existing sensors and measurement protocols against the goniometer, reporting promising results and significant reduction in measurement time, yet asking for further improvements in measurements protocols [24,27,33]. 

### 4.2. Leap vs. Goniometer

Most joints revealed minimal agreement between the results of the goniometer and the Leap motion sensor (Table 1). This may be explained by several different protocols were used to assess the finger ROM for each measurement technique. Using the goniometer, individual joint movements were assessed, whereas all fingers moved together for the assessment of finger flexion and extension during the Leap measurement. This may have resulted in some discrepancies between reachable angles, between the two techniques. However, we do not believe that the differences we found can be only attributed to this aspect. With the goniometer we measured the dorsal side of the hand and wrist (center of rotation outside of the joint), while the Leap motion sensor estimates the center of rotation inside the joint. In participants with protruding knuckles this can results in measurement differences between the two techniques. Moreover, while using the goniometer sometimes the rater is slightly pushing the measured joints and it is not clear to what extend this results in measurement of passive instead of active ROM. 

The disagreement in the results can also be attributed to the internal constraints of the Leap motion sensor. The system does not only rely on what the cameras can visualize, but also on the accuracy of the internal hand model. We noticed an increasing disagreement, while moving from proximal to more distal joints of the fingers. This can be attributed to the constraints of the Leap motion sensor, such as the coupling between the MCP and the DIP joint (θ_DIP_ = 2/3 θ_MCP_). This inherent coupling in the model, might explain the big disagreement for especially the thumb joints, between the two methods. In all cases, we noticed the participants’ thumb moving with a larger ROM in reality than the movement of the virtual thumb in the screen. Furthermore, the Leap motion sensor estimated rather than measure joint angles when occlusion was occurring. This may also explain the small standard deviations of the Leap results, whereas goniometer standard deviations were larger. Based on our results, we believe that the Leap motion sensor does not seem able to measure reliably at the extreme angle values for the measured joints (maximum and minimum angles). Since occlusion mainly occurred in the extreme flexion movements, the fact that we have measured maximum flexion and extension may have affected the results. This can also be observed in Figure 5c, where the difference between the two measurement techniques increases proportionally to the mean angle. Occlusion issues can be solved by using multiple Leap motion sensors. Placidi et al. [34] used 2 Leap motions sensors for tracking the position of the hand in 3D, resulting in reduced occlusions and without inducing further complications.

The use of goniometry and especially interrater assessments of finger ROM is also questionable with people suffering from hand related conditions, and would likely only produce less reliable measures [16]. The results of goniometric measurements indicate that it is difficult to show any change of a joint motion of less than 5° to 10° for most joints measured by the same tester [16]. Therefore, the Leap motion sensor should be evaluated as a viable alternative. 

### 4.3. Test vs. Re-Test

Regarding the test-retest reliability of the Leap motion sensor, we found a good agreement for all measured joints, except the DIP of the little finger. This result is probably due to the fact the Leap motion sensor, relies mostly to an estimation of the DIP rather than to the optical tracking. Most 95% CI have a span from 2° to 14°. This is similar to the reported intra-rater reliability of the goniometer, which is reported to be from 1.5° and up to 10° [15]. For the wrist measurements, we can see a lower agreement and larger 95% CI between test and retest measurements. Especially for pronation/ supination and wrist flexion/extension (Table 2). 

### 4.4. Time Consumption

Using the goniometer, the rater needed on average 32:65 min per participant. With the use of the Leap motion sensor, the rater was able to measure more joints at the same time, reducing measurement time to 7:22 min per participant. It can be assumed that more experienced raters can perform goniometric measurements in less time, however they would hardly be able to reach similar times to the Leap motion sensor measurements, without compromising accuracy. In addition, this study measured only one hand, while the Leap is able to measure two hands simultaneously, which could reduce the time consumption compared to the goniometer even further. In clinical practice, time is a very important aspect, as less time-consuming processes can allow the therapist to spend time with more patients, the patients to spend less time at the clinic and the overall costs to be reduced [35].

### 4.5. Lessons Learned

Regarding the wrist flexion/extension, we realized that the visibility of the elbow during the measurement, is important for the estimation of the wrist flexion/extension and pronation/supination angles. In our measurement protocol, due to the arm support (Figure 1) we used for our set-up, the elbow was occluded from the Leap motion sensor. Similarly, even after clear instructions to our participants to only pronate and supinate by moving their forearm, we believe that the large 95% CI is due to shoulder rotations during the assessment of this movement. More strict and uniform protocols can give more consistent and reliable measurements, regarding the wrist joint. This is currently also the case for the goniometer, where proper training and consistency in measurement technique are also important for therapists in order to perform reliable measurements [15].

### 4.6. Implications for Clinical Use

Although we have seen some clear advantages of using the goniometer over the Leap motion sensor there are also many advantages of using the Leap motion sensor over the goniometer, which could be especially useful in a clinical setting. Measurements with the Leap motion sensor are less time consuming and can also be used to assess dynamic and submaximal joint angles instead of only static and maximal joint angels. In addition, the Leap motion sensor is like the goniometer low-cost and no pre-calibration is required. Furthermore, no contact with the hand of the patient is required, which makes measuring with the Leap motion sensor less invasive, and possibly less painful than measurements with the goniometer. In addition, Leap motion measurements are in comparison to goniometer measurements much less dependent on the experience of the rater, which improves overall objectiveness and applicability of the method. Consequently, we think that the Leap motion sensor is a promising device for clinical use, but the applicability in people with limited hand function should still be investigated. The further development of the Leap motion sensor and future advances in technology can potentially offer a solution to occlusion and estimation of joint angles issues.

We believe that in the future, the Leap motion sensor can be used in combination with virtual or augmented reality together with gaming in order to motivate and enhance hand and wrist rehabilitation. This together with the ability of the Leap to measure joint angles at the same time, can enable evaluation of such futuristic interventions at the same time. Moreover, the low cost and the portability of the sensor, can allow the use of it for home rehabilitation and further reducing rehabilitation costs [35].

## 5. Conclusions

We performed an evaluation of the accuracy of the Leap motion sensor in comparison with the goniometer for 20 healthy participants. Additionally, we assessed the test-retest reliability of the sensor with 12 healthy participants. Our results give insight into the accuracy and reliability of this system and based on those results, we think the Leap motion sensor has potential to be used in clinical and research settings in the future. However, improvements have to be made in the measurement protocol and additional research is required to fully determine the optimal use of the Leap motion sensor. We were especially interested in the potential of the Leap motion sensor to evaluate the level of dysfunction and to guide adequate therapeutic strategies with people with DMD. Therefore, reliable assessment of hand function and joint ROM is important. At this point the Leap motion sensor cannot yet be used in this context, without further research in people with DMD. Future research should focus on the adjustment of the protocol in order to improve data acquisition and quality. Standardized protocols to set up and use the device must be established in order to ensure a reliable performance of the leap motion sensor, which will also add to the intra- and interrater reliability. Additionally, it is important to evaluate the performance of the Leap motion sensor for submaximal angles and assess the effect of the addition of an extra Leap motion sensor to solve occlusion issues. We believe that the current results contribute to further development of clinical protocols to use the Leap motion sensor in a clinical setting with patients.

## Figures and Tables

**Table 1:** Maximum joint angles measured with goniometer and Leap for all joints in all directions of movement. Means and standard deviations (sd) are given. Mean differences, 95% Confidence Intervals for the mean differences and the *p*-value for the one sample *t*-test of comparing the mean difference to zero are given. Bold letters indicate the joints and movements for which the two measurement techniques reached a good agreement. The Bland-Altman plots for these joints are illustrated in Figure 5.

Joint	Direction	Mean Gonio (sd)	Mean Leap (sd)	Mean Diff	95% CI of Mean Diff	One Sample *t*-Test (2-Tailed)
Wrist	Radial dev	19(4)	26(9)	−7	−11; −2	0.007
	**Ulnar dev**	**−35(5)**	**−38(6)**	**3**	**−1;6**	**0.115**
	Pronation	77(10)	90(12)	−13	−19; −7	<0.001
	Supination	−82(16)	−43(24)	−39	−51; −26	<0.001
	Flexion	79(8)	70(14)	9	4;14	0.001
	**Extension**	**−63(12)**	**−66(12)**	**3**	**−1;7**	**0.137**
Thumb MCP	Flexion	59(13)	36(6)	24	18;30	<0.001
	Extension	−5(15)	6(7)	−11	−17; −5	0.001
Thumb IP	Flexion	76(16)	28(4)	48	40;56	<0.001
	Extension	−25(6)	−2(8)	−23	−27; −18	<0.001
Index MCP	**Flexion**	**87(7)**	**88(2)**	**−1**	**−5;2**	**0.501**
	Extension	−18(9)	−11(8)	−7	−11; −3	0.003
Index PIP	Flexion	109(5)	82(2)	27	24;30	<0.001
	Extension	−3(4)	2(3)	−5	−8; −3	<0.001
Index DIP	Flexion	70(11)	43(9)	27	21;33	<0.001
	Extension	−12(6)	4(3)	−16	−19; −13	<0.001
Middle MCP	Flexion	90(6)	83(1)	7	4;10	<0.001
	Extension	−21(9)	−11(8)	−10	−14; −5	0.001
Middle PIP	Flexion	108(6)	85(2)	23	21;26	<0.001
	Extension	−4(6)	3(3)	−7	−10; −3	<0.001
Middle DIP	Flexion	81(8)	41(2)	39	36;43	<0.001
	Extension	−14(6)	4(4)	−17	−21; −14	<0.001
Ring MCP	Flexion	87(8)	80(1)	8	4;11	<0.001
	Extension	−23(9)	−11(9)	−12	−17; −7	<0.001
Ring PIP	Flexion	108(7)	81(2)	27	24;30	<0.001
	Extension	−6(10)	2(4)	−8	−14; −3	0.003
Ring DIP	Flexion	77(9)	40(1)	37	32;41	<0.001
	Extension	−15(8)	4(4)	−19	−23; −15	<0.001
Little MCP	Flexion	89(7)	71(2)	18	15;22	<0.001
	Extension	−27(13)	−14(6)	−13	−19; −6	<0.001
Little PIP	Flexion	100(6)	74(3)	26	23;29	<0.001
	Extension	−6(10)	2(3)	−8	−13; −4	0.001
Little DIP	Flexion	79(12)	40(2)	40	34;45	<0.001
	Extension	−11(9)	3(4)	−15	−19; −11	<0.001

**Table 2:** Test and retest maximum joint angles for all joints in all directions of movement. Means and standard deviations (sd) are given. Mean differences, 95% Confidence Intervals for the mean differences and the *p*-value for the one sample *t*-test of comparing the mean difference to zero are also reported. Bold letters indicate the joints and movements for which the test retest did not reach a good agreement.

Joint	Direction	Mean Leap Test (sd)	Mean Leap Retest (sd)	Mean Diff	95% CI of Mean Diff	One Sample *t*-Test (2-Tailed)
Wrist	Radial dev	27(9)	26(8)	1	−7;8	0.798
	Ulnar dev	−40(5)	−40(8)	0	−6;5	0.888
	Pronation	95(12)	99(15)	−4	−13;6	0.413
	Supination	−42(25)	−41(35)	−1	−23;21	0.939
	Flexion	75(11)	67(18)	8	−7;22	0.270
	Extension	−71(12)	−70(14)	−1	−10;7	0.724
Thumb MCP	Flexion	35(6)	34(5)	1	−3;4	0.757
	Extension	5(7)	6(8)	−1	−7;6	0.862
Thumb IP	Flexion	27(4)	25(3)	2	−1;6	0.214
	Extension	−3(8)	−5(9)	2	−5;9	0.507
Index MCP	Flexion	88(2)	88(2)	0	−2;2	0.891
	Extension	−8(7)	−9(5)	1	−2;4	0.537
Index PIP	Flexion	82(3)	81(3)	1	−1;3	0.380
	Extension	1(4)	0(2)	1	−2;4	0.467
Index DIP	Flexion	45(12)	41(2)	4	−3;11	0.249
	Extension	3(3)	2(2)	1	−1;4	0.198
Middle MCP	Flexion	83(1)	83(1)	0	−1;1	0.597
	Extension	−9(7)	−11(6)	2	−1;6	0.194
Middle PIP	Flexion	85(2)	84(3)	1	−1;4	0.255
	Extension	2(4)	1(2)	1	−1;4	0.300
Middle DIP	Flexion	41(3)	41(2)	0	−3;2	0.768
	Extension	3(4)	2(3)	1	−2;4	0.474
Ring MCP	Flexion	80(1)	80(1)	0	−1;1	0.884
	Extension	−8(9)	−12(6)	4	−2;9	0.183
Ring PIP	Flexion	81(2)	81(2)	0	−1;2	0.584
	Extension	2(5)	0(2)	2	−2;6	0.300
Ring DIP	Flexion	40(1)	41(1)	−1	−3;0	0.109
	Extension	3(4)	2(2)	1	−2;4	0.401
Little MCP	Flexion	71(1)	71(1)	0	−1;1	0.848
	Extension	−13(6)	−16(5)	3	−1;6	0.130
Little PIP	Flexion	75(3)	76(2)	−1	−3;0	0.122
	Extension	3(3)	1(2)	2	−1;4	0.183
Little DIP	**Flexion**	**39(2)**	**40(2)**	**−1**	**−2;−0**	**0.041**
	Extension	3(3)	2(2)	1	−1;3	0.389

## Supplementary Materials

Together with this manuscript, we also provide the data from our measurements. The supplementary file Data_Leap.xlsx contains all the data from the measurements with the Leap motion sensor and the goniometer. The data file is available online at http://www.mdpi.com/1424-8220/18/10/3230/s1.

## References

[1] Kapandji I.A. (1987). The Physiology of the Joints: Annotated Diagrams of the Mechanics of the Human Joints.

[2] Lutz W., Sanderson W., Scherbov S. (2008). The coming acceleration of global population ageing. Nature.

[3] Hung L.K., Ho K.K., Leung P.C. (1999). Impairment of hand function and loss of earning capacity after occupational hand injury: Prospective cohort study. Hong Kong Med. J..

[4] Bos R.A., Haarman C.J., Stortelder T., Nizamis K., Herder J.L., Stienen A.H., Plettenburg D.H. (2016). A structured overview of trends and technologies used in dynamic hand orthoses. J. Neuroeng. Rehabil..

[5] Schaechter J.D., Stokes C., Connell B.D., Perdue K., Bonmassar G. (2006). Finger motion sensors for fMRI motor studies. NeuroImage.

[6] Romitti P.A., Zhu Y., Puzhankara S., James K.A., Nabukera S.K., Zamba G.K.D., Ciafaloni E., Cunniff C., Druschel C.M., Mathews K.D. (2015). Prevalence of Duchenne and Becker Muscular Dystrophies in the United States. Pediatrics.

[7] Oess N.P., Wanek J., Curt A. (2012). Design and evaluation of a low-cost instrumented glove for hand function assessment. J. Neuroeng. Rehabil..

[8] Gajdosik R.L., Bohannon R.W. (1987). Clinical measurement of range of motion. Review of goniometry emphasizing reliability and validity. Phys. Ther..

[9] Mendell J.R., Lloyd-Puryear M. (2013). Report of MDA muscle disease symposium on newborn screening for Duchenne muscular dystrophy. Muscle Nerve.

[10] Bartels B., Pangalila R.F., Bergen M.P., Cobben N.A., Stam H.J., Roebroeck M.E. (2011). Upper limb function in adults with Duchenne muscular dystrophy. J. Rehabil. Med..

[11] Janssen M.M., Bergsma A., Geurts A.C., de Groot I.J. (2014). Patterns of decline in upper limb function of boys and men with DMD: An international survey. J. Neurol..

[12] Weichbrodt J., Eriksson B.M., Kroksmark A.K. (2017). Evaluation of hand orthoses in Duchenne muscular dystrophy. Disability Rehabil..

[13] Eagle M. (2002). Report on the muscular dystrophy campaign workshop: Exercise in neuromuscular diseases Newcastle, January 2002. Neuromuscul. Disord. NMD.

[14] Flextension Symbionics Project. http://symbionics.info/project3/.

[15] Lewis E., Fors L., Tharion W.J. (2010). Interrater and intrarater reliability of finger goniometric measurements. Am. J. Occup. Ther. Off. Publ. Am. Occup. Ther. Assoc..

[16] Bovens A.M., van Baak M.A., Vrencken J.G., Wijnen J.A., Verstappen F.T. (1990). Variability and reliability of joint measurements. Am. J. Sports Med..

[17] Norkin C.C., White D.J. (2009). Measurement of Joint Motion A Guide to Goniometry.

[18] Bassily D., Georgoulas C., Guettler J., Linner T., Bock T. Intuitive and Adaptive Robotic Arm Manipulation using the Leap Motion Controller. Proceedings of the 41st International Symposium on Robotics (ISR/Robotik 2014).

[19] Dipietro L., Sabatini A.M., Dario P. (2003). Evaluation of an instrumented glove for hand-movement acquisition. J. Rehabil. Res. Dev..

[20] Li K., Chen I.M., Yeo S.H., Lim C.K. (2011). Development of finger-motion capturing device based on optical linear encoder. J. Rehabil. Res. Dev..

[21] Gentner R., Classen J. (2009). Development and evaluation of a low-cost sensor glove for assessment of human finger movements in neurophysiological settings. J. Neurosci. Methods.

[22] Simone L.K., Kamper D.G. (2005). Design considerations for a wearable monitor to measure finger posture. J. Neuroeng. Rehabil..

[23] Kortier H.G., Sluiter V.I., Roetenberg D., Veltink P.H. (2014). Assessment of hand kinematics using inertial and magnetic sensors. J. Neuroeng. Rehabil..

[24] Pham T., Pathirana P.N., Trinh H., Fay P. (2015). A Non-Contact Measurement System for the Range of Motion of the Hand. Sensors.

[25] Bachmann D., Weichert F., Rinkenauer G. (2015). Evaluation of the Leap Motion Controller as a New Contact-Free Pointing Device. Sensors.

[26] Pambudi R.A., Ramadijanti N., Basuki A. Psychomotor game learning using skeletal tracking method with leap motion technology. Proceedings of the 2016 International Electronics Symposium (IES).

[27] Trejo R.L., Vázquez J.P.G., Ramirez M.L.G., Corral L.E.V., Marquez I.R. Hand goniometric measurements using leap motion. Proceedings of the 2017 14th IEEE Annual Consumer Communications & Networking Conference (CCNC).

[28] Guna J., Jakus G., Pogacnik M., Tomazic S., Sodnik J. (2014). An analysis of the precision and reliability of the leap motion sensor and its suitability for static and dynamic tracking. Sensors.

[29] Brekel. Affordable Motion Capture Tools—Pro Hands. https://brekel.com/brekel-pro-hands/pro-hands-download-trial-buy/.

[30] Bo A.P.L., Poignet P., Geny C. (2011). Pathological Tremor and Voluntary Motion Modeling and Online Estimation for Active Compensation. IEEE Trans. Neural Syst. Rehabil. Eng..

[31] Koo T.K., Li M.Y. (2016). A Guideline of Selecting and Reporting Intraclass Correlation Coefficients for Reliability Research. J. Chiropr. Med..

[32] Laerd Statistics. One-Sample T-Test Using SPSS Statistics. https://statistics.laerd.com/spss-tutorials/one-sample-t-test-using-spss-statistics.php.

[33] McVeigh K.H., Murray P.M., Heckman M.G., Rawal B., Peterson J.J. (2016). Accuracy and Validity of Goniometer and Visual Assessments of Angular Joint Positions of the Hand and Wrist. J. Hand Surg..

[34] Placidi G., Cinque L., Polsinelli M., Spezialetti M. (2018). Measurements by A LEAP-Based Virtual Glove for the Hand Rehabilitation. Sensors.

[35] Levanon Y. (2013). The advantages and disadvantages of using high technology in hand rehabilitation. J. Hand Ther. Off. J. Am. Soc. Hand Ther..

